# Effects of cinnamon essential oil and Persian gum on preservation of pomegranate arils

**DOI:** 10.1002/fsn3.2213

**Published:** 2021-03-18

**Authors:** Akbar Jokar, Hasan Barzegar, Neda Maftoon Azad, Maryam Shahamirian

**Affiliations:** ^1^ Agricultural Engineering Research Department, Fars Agricultural and Natural Resources Research and Education Center Agricultural Research, Education and Extension Organization (AREEO) Shiraz Iran; ^2^ Agricultural Sciences and Natural Resources University of Khuzestan Mollasani Iran

**Keywords:** anthocyanin, antimicrobial, ascorbic acid, coating, microbial spoilage

## Abstract

Given the high perishability of pomegranate arils, edible antimicrobial coating will enhance their shelf life and maintain their marketability. An antimicrobial coating was prepared using 1% (w/v) soluble part of Persian gum (PG) and different concentrations (0.25%, 0.50%, and 0.75% (v/v)) of cinnamon essential oil (CEO) to extend the shelf life of pomegranate arils. Microbiological, chemical, physical, and sensorial characteristics of coated and uncoated samples were evaluated at 7‐day intervals. Total anthocyanin (TAN), titrable acidity (TA), and ascorbic acid showed a decreasing trend, during the whole period of the storage. TAN, TA, and ascorbic acid decreased from 119.8 to 44.5 mg/L, 1.6% to 1.37%, and 682 to 140 mg/L, respectively. Firmness increased during the storage time, while total soluble solids (TSS, around 17.4 °Brix) and total phenolic compounds (TP, around 14.21 mg/100 ml) showed no significant changes with CEO concentrations. Coatings containing 0.5% and 0.75% CEO significantly prevented fungal growth on the samples at least for 3 weeks and 3 months, respectively. Optimization proved that 1‐week cold storage and 0.43% CEO could dramatically meet 80% of the research targets including maximum nutritional quality and freshness, as well preventing microbial spoilage. It was concluded that coating the pomegranate arils by PG and selecting an appropriate concentration of the CEO could considerably increase shelf life, marketability, and nutritional quality of pomegranate arils at a suitable and acceptable level.

## Graphical abstract

Coatings containing 0.5% and 0.75% cinnamon essential oil significantly prevented fungal growth on the pomegranate arils at least for 3 weeks and 3 months, respectively. Optimization proved that 1‐week cold storage and 0.43% CEO could dramatically meet 80% of the research targets including maximum nutritional quality and freshness, as well preventing microbial spoilage. Coating pomegranate arils by Persian Gum and selecting an appropriate concentration of the cinnamon essential oil could considerably increase shelf life, marketability, and nutritional quality of pomegranate arils at a suitable and acceptable level.

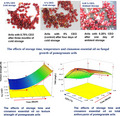

## 1 INTRODUCTION

Pomegranate is the fruit of *Punica Granatum* tree of *Punicacea* family. Iran is one of the largest pomegranate producers in the world, with a local area under extensive cultivation of more than 70,000 hectares and an annual production of about one million tons (Ahmadi et al., 2019). The extraordinary level of pomegranate cultivation and its increasing production typically induces us to devote particular attention to proper storage and postharvest effective control of contributing factors which naturally causing spoilage and waste. Recently, the use of ready‐to‐eat nutritious fruits and vegetables has been increased due to improving consumers' food habits.

Pomegranate arils (edible part of the fruit) have become enormously popular, as they are so wealthy in sugar, pectin, ascorbic acid, ellagic acid, amino acids, minerals, fibers, anthocyanin, phytoestrogens, and flavonoids. In this manner, it undoubtedly contains valuable materials positively influencing health (Martínez‐Romero et al., 2013; Oz & Ulukanli, 2012). As pomegranate arils are juicy with high water activity and rich in nutrients, the most important issue of them is their high microbial perishability. Pomegranate arils are highly susceptible to microbial spoilage by fungi and bacteria particularly yeast and mold growth (Caleb et al., 2015; Hussein et al., 2015; Kapetanakou et al., 2015).

There are several reports toward the importance of microbial spoilage of pomegranate arils and different preservation methods for controlling spoilage microorganisms. Some of the methods such as modified atmosphere packaging, high pressure, and chitosan coating were potent and could increase shelf life of the arils until 12–14 days in cold storage, while ultraviolet radiation, rinsing and chlorine disinfection, and different packaging materials showed no considerable and clear results (Ayhan & Eştürk, 2009; Banda et al., 2015; Bhatia et al., 2015; Caleb et al., 2015; Hussein et al., 2015; Kapetanakou et al., 2015; López‐Rubira et al., 2005; O'Grady et al., 2015).

In the studied preservation methods, the focus was mostly on the antioxidant activity and visual quality of the arils. These methods in some cases had negative effects such as chemical residuals, anthocyanin degradation, and limited inhibitory action against microorganisms, while all of the aspects of aril quality such as nutrition, appearance, taste, and microbial spoilage must be taken into the consideration (Ayhan & Eştürk, 2009; López‐Rubira et al., 2005).

Edible films and coatings, which especially executed with gum, could maintain the quality and shelf life of food products by regulating the transfer of moisture, oxygen, carbon dioxide, aroma, and flavors (Bagheri et al., 2016; Gardesh et al., 2016; Javadian et al., 2017; Moalemiyan et al., 2012; Villafañe, 2017).

The term “gum” describes a class of naturally occurring carbohydrates, which are usually water‐soluble and are used for various applications such as coating. Edible coatings based on gums create an especial atmosphere around the fruit or vegetable by providing a semi permeable membrane to CO2 and O2, thus controlling respiration and oxidation reaction rates. Gums have many advantages over the other coating agents such as synthetic waxes, as they have been recognized as GRAS by FAO, and their application is safe for the consumer and human health, renewable, economically, and eco‐friendly (Khorram et al., 2017; Salehi, 2020; Tahir et al., 2019).

Researchers identified and utilized various types of gums as coating agents for preserving fruits and vegetables. There are lots of reports for coating fruits and vegetable with gums as a preserving method: coating fresh cut apple with gellan gum (Moreira et al., 2015), fresh cut papaya with Psyllium (Plantago) (Yousuf & Srivastava, 2015), carrot with alginate gum (Amanatidou et al., 2000), mango with Arabic gum (Khaliq et al., 2015), apricot with basil seed gum (Hashemi et al., 2017), and many others (Salehi, 2020; Tahir et al., 2019).

Persian gum is typically a natural gum or hydrocolloids exudate from *Amygdalus Scoparia* tree (Abbasi et al., 2011). Researchers showed that the low surface tension of PG demonstrates its potential to be used as a suitable, new, and applicable coating material for cucumber and tomato fruits (Mostafavi, 2019). Khorram et al. used PG as a coating agent on “Valencia” oranges and announced that this edible coating could decrease water loss and softening of oranges during 60 days cold storage (Khorram et al., 2017). Shaygannia et al. used PG in combination with other gums as a gelling matrix for encapsulation of lemon waste extract. The results proved that encapsulation of functional substances from lemon waste extract such as phenolic compounds by PG could effectively protect them in dark and light conditions (Shaygannia et al., 2020).

Coating of pomegranate arils with chitosan caused more preservation of TP and anthocyanin, anti‐oxidation properties and vitamin C (Zahran et al., 2015). Coating of pomegranate arils with aloe vera gel diminished fungi and aerobic mesophilic microorganisms, preserved texture strength, and increased anthocyanin and phenolic materials of the arils as well as maintaining sensorial properties. Anthocyanin and color Chroma (saturation) decreased for all uncoated and coated Pomegranate arils with storage time, while lightness and hue angle increased. These negative changes considerably reduced in coated samples with chitosan (1% and 2%) and at lower storage temperatures (Varasteh et al., 2012). These dada proves that coating could be a promising method to prolong the shelf life of pomegranate arils.

Applying a combination of essential oils and gums as a coating can improve the shelf life and quality of foods. There are many reports related to combination of coating materials with essential oils for preservation of foods especially fruits and vegetables. Eryngium campestre essential oil incorporated in chitosan nanoparticles for coating cherry fruits (Arabpoor et al., 2021). Essential oils of cinnamon, basil, and thyme combined with sodium alginate for peach (Ayub et al., 2020). Chitosan contained Cymbopogon citratus Stapf essential oil for coatings guava (de Oliveira et al., 2020). Thyme essential oil was loaded in electrospinning porous poly lactic acid nanofibers for preserving strawberry (Min et al., 2021). Citronella essential oil incorporated in chitosan nanocomposite films in order to prolong the shelf life of coated grapes (Motelica et al., 2020). Clove essential oil in chitosan for coating apple fresh cuts (Wang et al., 2021).

Cinnamon essential oil generally represents a potent antimicrobial agent (Burt, 2004). The effective antimicrobial compound in CEO is cinnamon aldehyde, which is very efficient in reducing viable fungi and bacteria in fruits especially with low pH (3.2–3.6) such as pomegranate arils (Roller & Seedhar, 2002). Cinnamon aldehyde binds to the proteins in the cell walls of microorganisms and inhibits amino acid decarboxylase enzymes (Burt, 2004).

With increasing demand for fresh and natural preservatives, antimicrobial coatings which contain essential oils seems to be an ideal and new alternative for preservation of minimally processed pomegranate arils (Frazao et al., 2017; Ghasemnezhad et al., 2013; Gniewosz et al., 2013; Martínez‐Romero et al., 2013).

In this academic study, pomegranate arils coated with PG combined with CEO, and the potential effects of PG and CEO concentrations on the physicochemical properties of arils in ambient and cold storage were carefully investigated. There are not any reports in literatures giving data for shelf life of pomegranate arils in ambient temperature. As the pomegranate arils might be remained in ambient temperature for a while, therefore studying the life span of the arils in ambient could be interesting, useful and informative. To our extensive knowledge, no similar work has been done before.

## 2 MATERIALS AND METHODS

### 2.1 Materials

Cinnamon essential oil was purchased from Zard Band Company. Cumin oil was prepared by the cold press method. Folin–Ciocalteu, 2, 6‐dichloroindophenol and ascorbic acid prepared from Scharlau Company. Sodium carbonate, methanol, acetone, sodium hydroxide, sodium acetate, and hydrochloric acid were supplied from Merck Company.

### 2.2 Methods

#### 2.2.1 Preparation of pomegranate arils

Rabab pomegranates were carefully prepared from local Ghasroddasht gardens, Shiraz, Iran, and properly stored at 5°C. Pomegranate arils removed manually from the skin and internal parts, immediately divided into equal portions, and immersed in coating solutions.

#### 2.2.2 Collection and grinding of PG

In August and September 2018, gums exuded by *Amygdalus Scoparia* trees were collected in Pars Province, Iran. Due to the significant diversity of PG (white to brown), only pearly white gums were carefully selected for this study. GP milled by an electrical mill (Retsch) and finally was sieved (by mesh 60) to obtain a fine and homogenous powder (Rahimi et al., 2013).

#### 2.2.3 Separation of the soluble part of PG

Separation of the soluble part of PG was conducted according to Samari‐Khalaj and Abbasi (2017) with slight modification. PG powder was dissolved in distilled water (1% w/v) using a magnetic mixer to separate the soluble part of the gum. The resultant solution was kept at ambient temperature for 24 hr to complete the soaking process. Then, the gum solution was centrifuged (SORVALE Super Speed, RC_2_‐B) at 33,000 *g* for 15 min. Finally, the supernatant containing the soluble part of the gum was separated and applied for coating (Samari‐Khalaj & Abbasi, 2017).

#### 2.2.4 Pomegranate aril coating

In our previous work, the possible effects of different amounts of PG coating (0%, 1%, and 2% of PG) on the pomegranate arils were properly investigated. The results and analysis cannot be presented here, as they are lengthy and sophisticated. According to the key findings and reasonable conclusions, the most suitable concentration for coating the arils efficiently was 1% PG. Therefore, in the present research, we exclusively use 1% PG.

Firstly, adequate portions of PG solution in 1% concentration of soluble part, along with 20% cumin oil (as plasticizer), were prepared. As it has been proved in previous literatures that Nigella sativa oil had antimicrobial effects (Oz & Ulukanli, 2012), cumin oil was applied in this study; however, in present research it did not show any significant antimicrobial effect. In the second step, for producing specific combinations of PG and CEO solutions, various contents of CEO (0%, 0.25%, 0.5%, and 0.75% (v/v)) were added to the PG solutions from the first step. These treatments were selected according to our previous study and also other scientific reports (Ayub et al., 2020; Barzegar et al., 2018; Wang et al., 2021). Next, the solutions were fully emulsified by a homogenizer (WTW‐Disper) at 12,000 rpm for 5 min. The pomegranate arils were dipped in the prepared coating solutions for 10 min. Then, the coated arils were gently spread on the clean metal nets, exposed to the fresh air for 5 hr to be dried naturally. Eventually, 200 g of the arils were packed in polypropylene containers for each treatment (Caleb et al., 2015).

For chemical experiments and juice extraction, pomegranate seeds were crushed for 20 s with a mixer (Bosch, MMB65G5M) and then the obtained juice was sufficiently separated with a metal fine mesh.

#### 2.2.5 Titratable acidity (TA)

Aliquots (2 ml) of pomegranate juice was titrated with 0.1 N sodium hydroxide to an endpoint of pH = 8.2 (Banda et al., 2015). Equation 1 calculated the TA:

(1) $$\text{TA}\%\left(\text{as}\;\text{citric}\;\text{acid}\right)=\frac{\left(V\times 0.1\times 0.064\times 100\right)}{M}$$

where *V* denotes milliliter of the sodium hydroxide and *M* is the volume of the juice sample.

#### 2.2.6 Total anthocyanin (TAN)

The method of pH differential, with potassium chloride (pH = 1) and sodium acetate (pH = 4.5) buffers, was employed to measure TAN of the samples (Frazao et al., 2017). One milliliter of juice of the samples was added to 14 ml of ethanol (50% v/v). Then, it was centrifuged at 4,000 *g* for 15 min. One milliliter of the supernatant was added to 7 ml of each buffer, separately. After 10 min, using a visible spectrophotometer (Pharmacia Biotech), absorptions were measured at 510 and 700 nm for each buffer, individually. Equation 2 calculated A (absorption difference) of the samples:

(2) $$A={\left(A_{510}‐A_{700}\right)}_{\text{pH}\;1.0}‐{\left(A_{510}‐A_{700}\right)}_{\text{pH}\;4.5}$$

In which, *A*_510_ and *A*_700_ were the absorptions at 510 and 700 nm, respectively. Then, Equation 3 calculated the TAN of each sample:

(3) $$\text{TAN}=\frac{\left(A\times \text{MW}\times \text{DF}\times 100\right)}{\text{MA}\times L}$$

where MW is the molecular weight of pelargonidin 3‐glucoside (443), DF is the dilute factor, MA is the molar extinction coefficient of pelargonidin 3‐glucoside (15,600), and *L* is the cell length (1 cm).

#### 2.2.7 Total phenolic compounds (TP)

Fifty milliliter of methanol–acetone–water (with the same portions) solution was added to 5 ml of each juice sample, following centrifugation at 4,000 *g* and 25°C for 10 min. TP was measured by Folin–Ciocalteu solution according to Slinkard and Singleton's method (Slinkard & Singleton, 1977). The gallic acid standard curve was used for the calculation of TP. The results were reported as mg of Gallic acid in 100 ml of pomegranate juice.

#### 2.2.8 Total soluble solids (TSS)

Total soluble solids (°Brix) was measured by a handy refractometer (ATAGO HSR500). TSS is a suitable index for total sugar content in fruit juices (Caleb et al., 2015).

#### 2.2.9 Ascorbic acid

Ascorbic acid was determined by 2,6‐dichlorophenolindophenol colorimetry method, according to Barros et al. (2007) with slight modification. One milliliter of pomegranate juice was added to 10 ml of metaphosphoric acid (1% w/v) and centrifuged for 15 min at 4,000 *g*. One milliliter of supernatant was added to 10 ml 2,6‐dichlorophenolindophenol (0.0025% w/v) and the solution was kept for 10 min at the dark, the absorbance of the samples was measured at 515 nm, after 10 min being kept at dark. Ascorbic acid contents were calculated based on a calibration curve of origin L‐ascorbic acid solutions (0–100 mg/L) (Barros et al., 2007).

#### 2.2.10 Texture strength

The texture strength of the pomegranate arils was determined by a digital penetrometer (TR Faccini TPA, Copernico‐Italy Company). For this purpose, a flat cylindrical probe with an 8 mm diameter and a speed of 2.5 mm/s was employed. The maximum force (N) for the rupture of the arils surface was reported (Ayhan & Eştürk, 2009; Kumar et al., 2016).

#### 2.2.11 Microbial spoilage

The microbial spoilage was investigated by observing the arils and daily recoding the number of spoiled arils. It was not possible to perform a microbiological culture test since even with one spoiled pomegranate aril; the entire surface of the plate was covered with fungal mycelium (Uncountable test). The dilution counting method was not also applicable to fungal growth enumeration. Therefore, we have accurately reported the direct results as percent of spoiled arils in each applied treatment.

#### 2.2.12 Sensorial evaluations

After 3 weeks of storing the samples in the refrigerator, sensorial evaluations were performed on three selected samples: fresh pomegranates arils as control and coated arils containing 0.50% and 0.75% of CEO. Sensorial evaluations were conducted using the 5‐point hedonic scaling test by 20 semi‐trained panelists (Lawless & Heymann, 2010).

#### 2.2.13 Statistical analysis

Experiments were conducted in a factorial arrangement with two factors: time (1, 2, and 3 weeks) and CEO concentration (0%, 0.25%, 0.50%, and 0.75%) in a completely randomized design (CRD) with two replications. ANOVA was performed with SPSS version 20 software and mean comparison was done by Duncan test. The nonparametric method was applied for sensorial analysis: Kruskal–Wallis and Mann–Whitney tests by SPSS version 20. The statistical software calculated and reported the mean rank of the selected samples for each sensorial property.

#### 2.2.14 Control sample

On the fourth day of cold storage (5°C), fungal mycelia were seen clearly on the arils' surface in the control samples (Arils with 0% CEO and 1% PG). Therefore, the control sample became completely unevaluable. The results of such a sample were entirely unacceptable, and it was impossible to include it in the analysis with other treatments. Therefore, the control sample removed from the statistical comparison and the other treatments were analyzed and compared.

#### 2.2.15 Models and optimization

Design Expert (version 7.0) was applied for developing models and optimization. Using numerical optimization, goals and importance level for main properties were set (Table 2), and finally optimum treatment was introduced by the software.

## 3 RESULTS AND DISCUSSION

### 3.1 Titratable acidity

**FIGURE 1:**
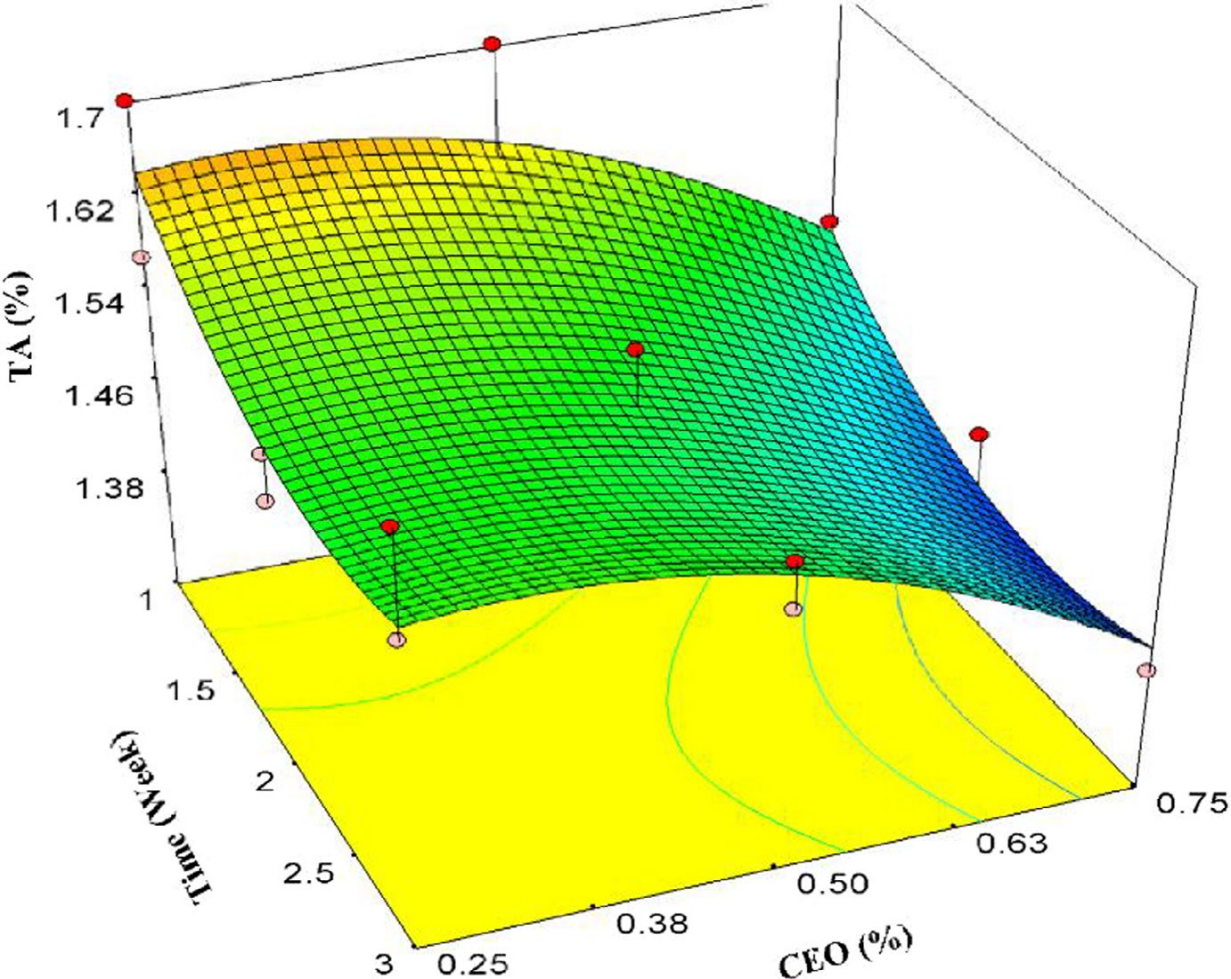
Effects of storage time and CEO content on TA

Figure 1 shows that TA decreased during cold storage compared with the initial day and the first week of the experiment. TA increased slightly by increasing CEO concentration until 0.5%, while it decreased at 0.75% concentration, significantly (Figure 1). Changes in TA followed a quadratic model Proper model for TA were quadratic (Equation 4). According to ANOVA the model, time and CEO were significant (*p* < .01) while time^2^, CEO^2^, and lack of fit were not significant (*p* > .05). Adequate precision, which indicates a signal to noise ratio, was 8.808. The ratio greater than four is desirable.

(4) $$\begin{array}{c} \text{TA}=+1.76‐0.25\times t+0.55\times \text{CEO}+0.052\times t^{2}‐0.85\times {\text{CEO}}^{2}\\ \begin{array}{ccc} R^{2}=.76 & \text{Adj}\;R^{2}=.68 & \text{Adequate}\;\text{Precision}=8.808 \end{array} \end{array}$$

*t represents the time of storage in all of the equations.

Titrable acidity reduction is attributed to the metabolism and respiration of the pomegranate arils, such that the acidic substances participate in a series of biochemical reactions and converts to other nonacidic compounds. Similar results were reported by other researchers (Ayhan & Eştürk, 2009; Banda et al., 2015; Oz & Ulukanli, 2012). On the contrary, to the results of this study, there are some reports about a slight increase in pomegranate TA, particularly in the first days of storage (Ghasemnezhad et al., 2013; Jiang & Li, 2001). Martínez‐Romero et al. (2013) reported a decline of TA in pomegranate arils over time, but they announced that coating the pomegranate arils with aloe vera gel, containing citric and ascorbic acid, increased TA. Although we expected, increasing the CEO content could result in preserving higher TA, our findings were opposite specifically at 0.75% CEO concentration (Figure 1). It might be due to the reactions between the active substances from CEO and acidic compounds in the pomegranate arils.

### 3.2 Total anthocyanin

**FIGURE 2:**
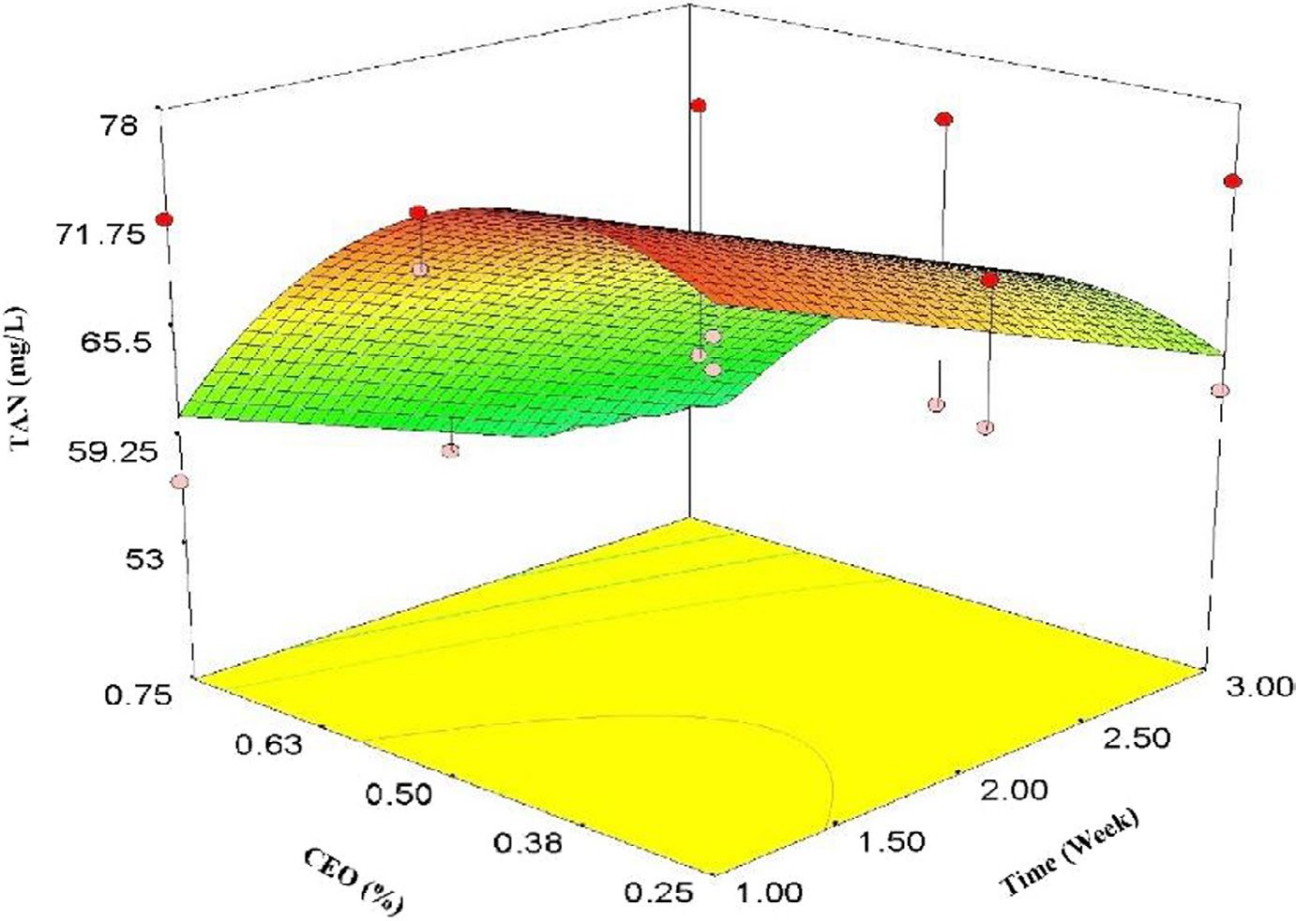
Effects of time and CEO on TAN

Figure 2 represents the effects of time and CEO concentrations on TAN. Compared with the first day, TAN considerably declined after 1 week and this trend continued until the end of the third week (*p* < .05). As the same as TA, increasing CEO concentration from 0.25% to 0.5% caused an increment of TAN, while a significant reduction can be seen at 0.75% concentration (Figure 2). Oxidation of the pomegranate arils during storage deteriorated TAN. The best model for TAN is presented in Equation 5. According to ANOVA quadratic model, time, CEO, and CEO^2^ were significant (*p* < .01), while time^2^ and lack of fit were not significant (*p* > .05).

(5) $$\begin{array}{c} \text{TAN}=+63.78‐5.57\times t+98.27\times \text{CEO}‐126.77\times {\text{CEO}}^{2}\\ \begin{array}{ccc} R^{2}=.63 & \text{Adj}\;R^{2}=.55 & \text{Adequate}\;\text{Precision}=7.755 \end{array} \end{array}$$

Cinnamon essential oil concentrations influence the TAN significantly, such that 0.75% of CEO led to a remarkable reduction of TAN compared with 0.25% CEO. Different constituents of CEO, specifically cinnamon aldehyde, might have affected TAN and caused the reduction of this colorful and valuable compound especially at the higher concentrations of CEO. Further studies are suggested to clarify the influence of CEO on TAN content.

Total anthocyanin reduction because of passing storage time was also reported by other researchers (Bhatia et al., 2015; Dokhanieh et al., 2016). The main reason for TAN reduction in pomegranate arils could be related to increasing respiration rate due to the stress and damage of initial processing such as separating the arils from pomegranate peels, moisture content reduction, changes of acidity, and TSS. Moreover, pH and the structure of the fruits and vegetables play important roles in the stability of TAN (Bhatia et al., 2015). However, Oz and Ulukanli (2012) reported that TAN content increased, in pomegranate arils coated by starch‐containing *Nigella Stavia* oil.

### 3.3 Total phenolic compounds

Total phenolic compounds content did not change significantly by storage time and CEO concentrations. No remarkable difference in TP was observed by the interaction of different storage times and CEO concentrations. The lack of fit for this model was significant; therefore, no graph was depicted for the influence of storage time and CEO on TP.

On the first day, TP content was 14.21 mg/100 ml (mg Gallic acid per 100 ml of pomegranate juice) and it did not change until the end of the experiment. Many researchers reported a reduction of phenolic substances for pomegranate and other fruits during storage (Dokhanieh et al., 2016; Martínez‐Romero et al., 2013; Palma et al., 2015). We have also expected TP reduction in the present research, but it did not happen. This result might have been due to the prevention of TP deterioration by PG coating and CEO. In support of this result, Ghasemnezhad et al. (2013) reported that coating pomegranate arils with chitosan diminished phenol oxidase enzymes and enhanced phenolic substances (Ghasemnezhad et al., 2013). Martínez‐Romero et al. (2013) announced that phenolic substances, in coated pomegranate arils with aloe vera, increased until 12 days and then reduced. Furthermore, the inclusion of citric and ascorbic acid to the aloe vera gel enhanced phenolic compounds, while the aloe vera gel could not prevent the reduction of phenolic substances by itself (Martínez‐Romero et al., 2013).

### 3.4 Total soluble solids

**FIGURE 3:**
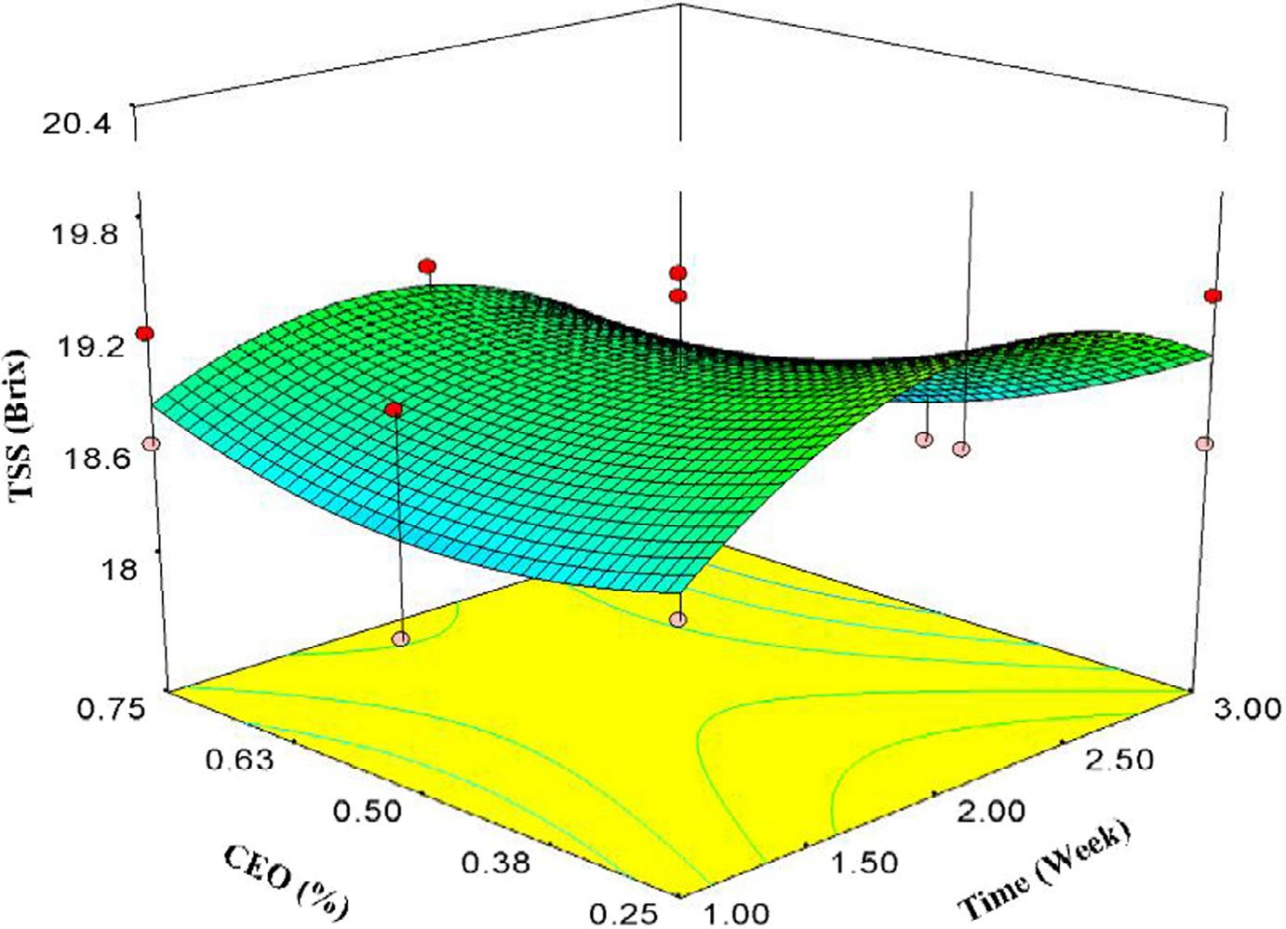
Effects of time and CEO on TSS

Storage time and CEO concentration had no significant effect on TSS of the arils (Figure 3). In other words, the TSS fluctuations did not have a regular and scientific trend. Therefore, no proper model could predict the TSS changes with reasonable *R* ^2^.

Total soluble solids content changed from 17.4% on the first day to 19% after 3 weeks. TSS increment by increasing storage time might be attributed to the moisture and weight loss of the arils during cold storage. Several researchers reported the same results in pomegranate arils and other fruits (Ghasemnezhad et al., 2013; Gil et al., 1996; Oz & Ulukanli, 2012). There are some reports in favor of TSS reduction in pomegranate arils (Artés et al., 2000; Ayhan & Eştürk, 2009; Banda et al., 2015).

Total soluble solids reduction might be related to the metabolic reaction and respiration of the arils causing more utilization of sugars and consequently, TSS to be decreased (Ghasemnezhad et al., 2013; Jiang & Li, 2001; Oz & Ulukanli, 2012; Peña‐Estévez et al., 2016). In support of the results of this study, Palma et al. (2015) declared that TSS did not change significantly (Palma et al., 2015). The reasons for these different results might be due to the application of various treatments to the pomegranate arils such as packing, storage conditions, and pomegranate varieties (Palma et al., 2015).

Total soluble solids reduction (in some cases) might be related to metabolic reactions and respirations of the arils. These reactions consume TSS, usually sugar, and as a result, TSS will be reduced (Ghasemnezhad et al., 2013; Jiang & Li, 2001; Oz & Ulukanli, 2012; Peña‐Estévez et al., 2016).

### 3.5 Ascorbic acid

**FIGURE 4:**
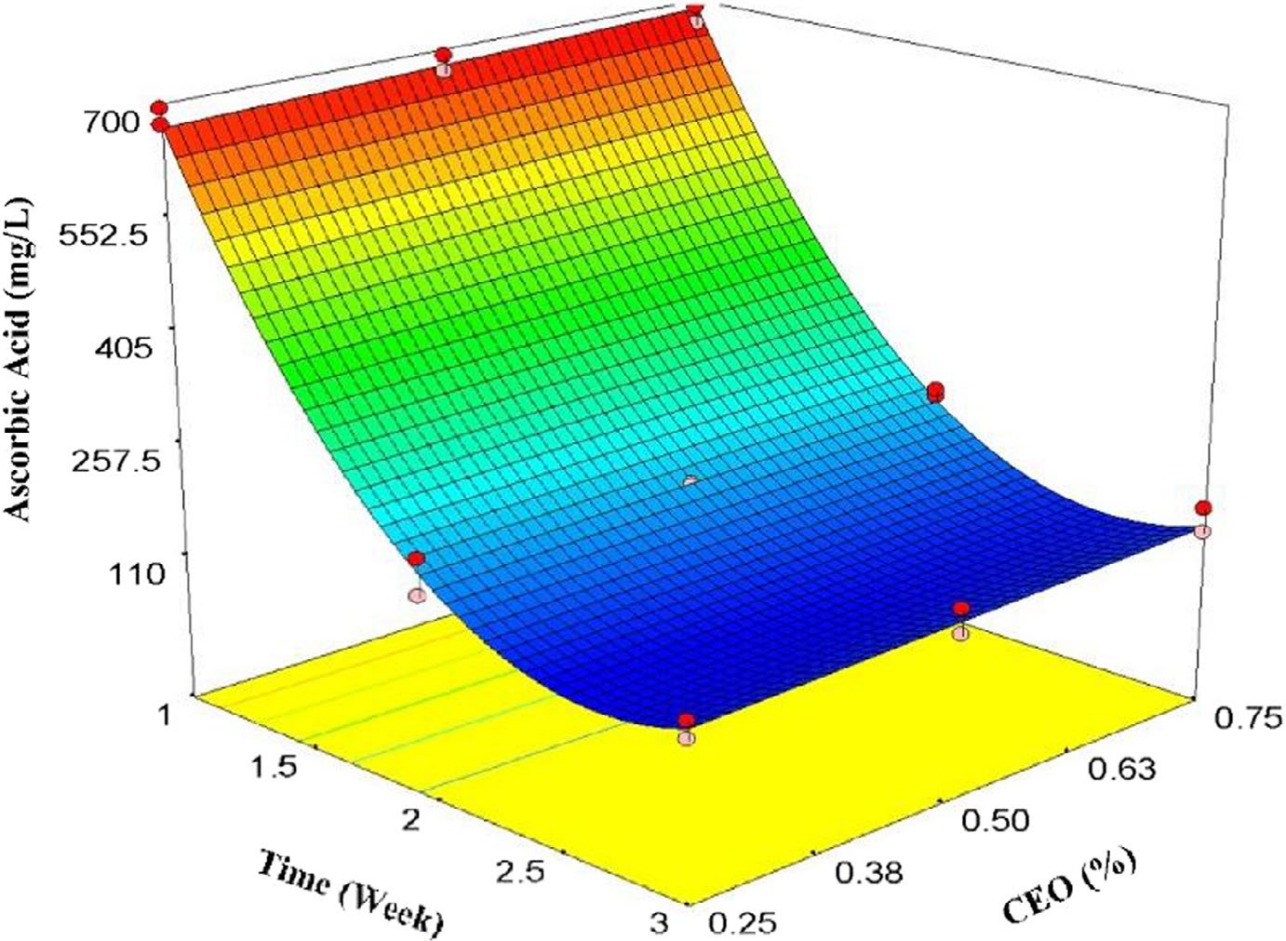
The effects of time and CEO on ascorbic acid

As it is depicted in Figure 4, ascorbic acid content decreased drastically in the second and third weeks of cold storage. Therefore, the effect of time on ascorbic acid was noticeably more than coating and CEO concentration. A quadratic model was well fitted to this change (Equation 6). The quadratic model, time, and time^2^ were significant (*p* < .01), while CEO, CEO^2^, and lack of fit were not significant (*p* > .05).

(6) $$\begin{array}{c} \text{Ascorbic}\;\text{Acid}=+1530.69‐1071.59\times t+38.94667\times \text{CEO}+200.95167\times t^{2}\\ \begin{array}{ccc} R^{2}=.996 & \text{Adj}\;R^{2}=.995 & \text{Adequate}\;\text{Precision}=68.334 \end{array} \end{array}$$

Oxidation of ascorbic acid was the main reason for this tremendous reduction. At the beginning of the first week of storage, the ascorbic acid content was measured as 682 mg/L, while it decreased to 140 mg/L in the third week of storage. The reduction of ascorbic acid during storage was reported by other researchers (Bhatia et al., 2013, 2015; Dokhanieh et al., 2016; Zahran et al., 2015). Moisture loss, damage to the cell walls, storage conditions (moisture and temperature), and different packaging could be the reasons for this remarkable reduction.

### 3.6 Texture strength

**FIGURE 5:**
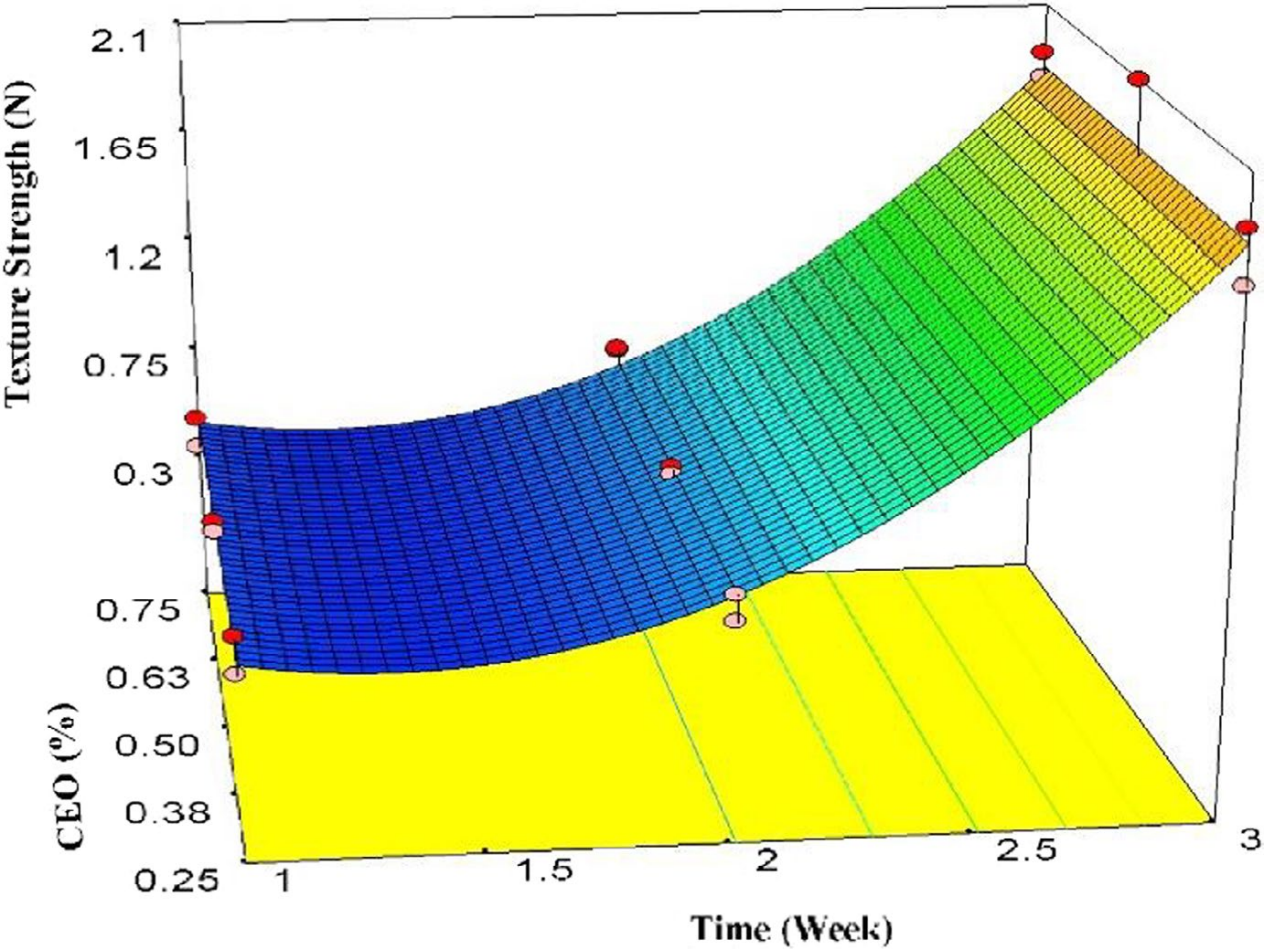
The effects of time and CEO on texture strength

As shown in Figure 5, arils' texture strength increased over time, specifically at the end of the third week of storage (*p* < .05). However, compared with the first day, texture strength showed a decreasing trend in the first week. Figure 5 clearly shows no significant effect of CEO concentration on texture strength. The model for texture strength is depicted in Equation 7. ANOVA showed that the effects of the quadratic model, time, time^2^ were significant (*p* < .01); however, CEO, CEO2, and lack of fit were not significant in the model (*p* > .05).

(7) $$\begin{array}{c} \text{Texture}=+1.27‐1.33\times t+0.5\times t^{2}\\ \begin{array}{ccc} R^{2}=.982 & \text{Adj}\;R^{2}=.971 & \text{Adequate}\;\text{Precision}=33.273 \end{array} \end{array}$$

On the first day, the required force for collapsing the surface of pomegranate arils was 0.59 N, but it reached 1.85 N after 3 weeks of storage. Increasing texture strength or prevention of softening pomegranate and other fruits including sweet and sour cherry, table grape, lime fruit, strawberry, papaya, and nectarine by coating were also reported by other researchers (Maftoonazad & Ramaswamy, 2019; Martínez‐Romero et al., 2013; Oz & Ulukanli, 2012). According to these researchers, the main reasons for increasing texture strength are (a) prevention of moisture loss by the coatings, (b) lowering respiration and metabolism, and (c) delay in ripening. These findings are in line with the present study. We believe that a slight reduction in the moisture content of the coating could lead to a physical change on the arils' surfaces following an increment in the elasticity or reduction of arils' brittleness. Therefore, more force was required to collapse the surface of the coated pomegranate arils.

On the contrary, some researchers reported texture strength depletion for pomegranate arils and fresh cut pears during storage (Bhatia et al., 2013; Soliva‐Fortuny et al., 2002). They concluded that aerobic metabolism, moisture loss, stress, or damage to the fruits during processing and handling led to increasing cell wall enzyme activity, pectin decomposition, and finally softening fruit structure. While in the present study and other similar works, softening factors have been extensively controlled by the coating and use of cold storage.

### 3.7 Microbial perishability

**FIGURE 6:**
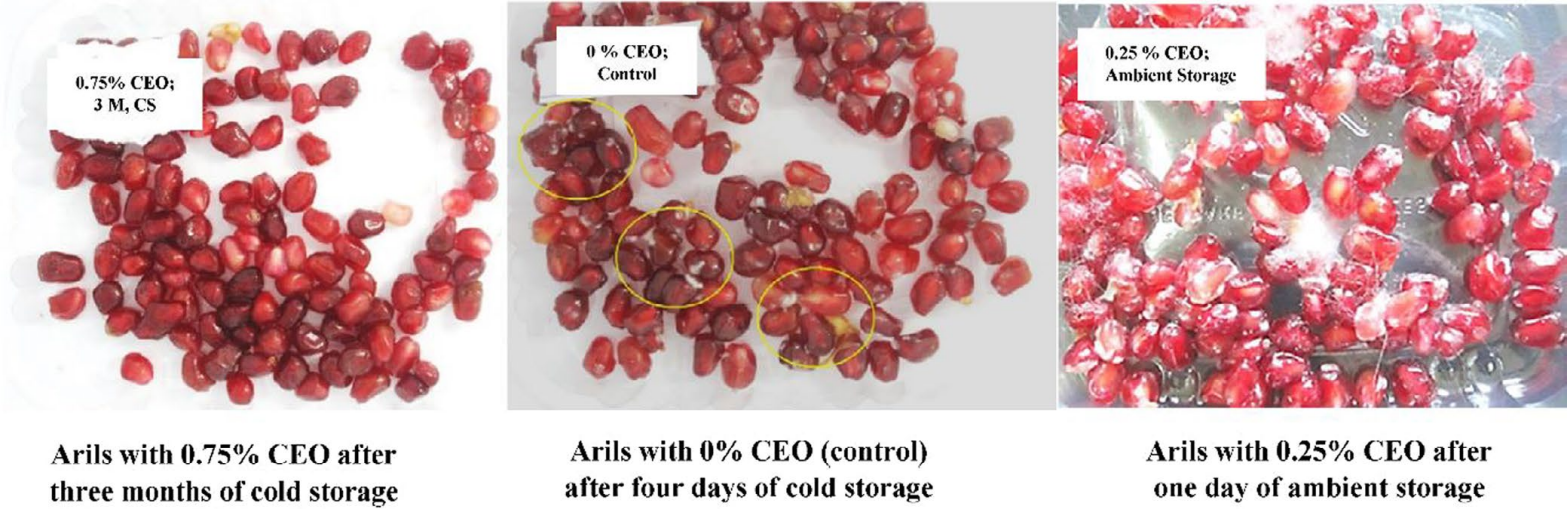
Fungal growth on the arils in different treatments

Fungal growth and their mycelia were obviously seen (80% spoilage) on the coated arils with 0.25%, 0.5%, and 0.75% of CEO after 24, 48, and 72 hr at ambient storage, respectively. The shelf life of 2 or 3 days represents an outstanding success for the pomegranate arils because the coated arils were packed and kept at ambient temperature. Arils in the control sample were completely covered with fungal mycelia (90% spoilage) after 24 hr at ambient temperature (Figure 6).

On the fourth day of cold storage (5°C), fungal mycelia were seen clearly on the arils' surface in the control samples around 50% spoilage. Coated samples with 0.25% CEO were spoiled on the fourth week and fungal mycelia covered 20% of the arils. Nearly 20% of the arils coated by 0.5% CEO spoiled after 6 weeks of cold storage. Finally, coated arils with 0.75% CEO remained fresh and unspoiled until 3 months with no visible signs of fungal growth (Figure 6).

### 3.8 Sensorial evaluation

The analysis of sensorial evaluations is presented in Table 1. The statistical analysis showed no significant difference between the tastes of different samples (*p* > .05). The taste of cinnamon was easily felt from the coated samples, but their acceptability was similar to the control. The most important reason for using essential oils is to improve the antimicrobial and anti‐oxidation properties of hydrophilic coatings (Martínez‐Abad et al., 2013). In addition to these positive effects, undesired sensorial effects may be created in the coatings because of the presence of aromatic and volatile compounds from essential oils. In the present study, CEO has not created any undesired smell or taste in the coated samples.

**TABLE 1:** The results of the sensorial analysis of pomegranate arils

Samples	Mean rank of taste score	Chi‐statistics	Significance
Control pomegranate arils	31.58		
Pomegranate arils with 0.5% of CEO	29.43	0.169	0.919
Pomegranate arils with 0.75% of CEO	30.50		

### 3.9 Optimization

The specific targets of the present study were preserving pomegranate seeds against microbial spoilage, retaining freshness, and valuable nutritional compounds (TAN and Ascorbic acid). According to the mentioned purposes of this study, the optimum conditions for preserving pomegranate seeds were as 1‐week cold storage and 0.43% CEO (Table 2). Using this treatment (1‐week cold storage and 0.43% CEO), 80.1% of the purposes were covered. However, according to the performed numerical optimization, increasing the time of storage, for instance, 2 weeks of cold storage reduced desirability up to 40%. In other words, 50% of the overall quality and especially nutritional aspects of the arils decreased in direct comparison with 1‐week storage.

**TABLE 2:** Optimization conditions and selected sample

Name	Goal	Lower limit	Upper limit	Importance	Selected sample with 80.1% desirability
Time	Is in range	3	1	3	1 (week)
CEO	Is in range	0.75	0.25	3	0.43 (%)
Texture	Is in range	2.1	0.34	2	0.45 (N)
Moisture	Maximize	77.29	75.3	3	76.54 (%)
TA	Is in range	1.7	1.38	3	1.64 (%)
TP	Is in range	16.95	12.77	5	14.22 (mg/100 ml)
TAN	Maximize	77.96	37.63	5	77.04 (mg/L)
Ascorbic acid	Maximize	692.41	119.7	5	676.74 (mg/L)
a	Maximize	43.5	30	3	37.23

## 4 CONCLUSION

Coating pomegranate arils with PG and CEO controlled and inhibited microorganisms intensively, even at ambient temperature. The influence of CEO was more significant than that of PG. Increasing storage time reduced the amounts of some valuable nutrients, such as ascorbic acid and TAN. However, phenolic substances remained almost unchanged. Different concentrations of CEO and storage time had no negative effects on the color, texture, and sensorial properties of the pomegranate arils. CEO could prevent the growth of fungi considerably, especially at a concentration of 0.75%. By coating the pomegranate arils and selecting an appropriate concentration of CEO, shelf life, marketability, and nutritional quality of pomegranate arils can be controlled and preserved at an acceptable level.

## CONFLICT OF INTEREST

The authors declare that they do not have any conflict of interest.

## ETHICAL APPROVAL

This study does not involve any human or animal testing.

## INFORMED CONSENT

Written informed consent was obtained from all study participants.

## Data Availability

The data that support the findings of this study are available from the corresponding author upon reasonable request.

## References

[fsn32213-bib-0001] Abbasi, S. , Mohammadi, S. , & Rahimi, S. (2011). Partial substitution of gelatin with persian gum and use of olibanum in production of functional pastile. Iranian Journal of Biosystem Engineering, 42(1), 121–131.(In Persian).

[fsn32213-bib-0002] Ahmadi, K. , Gholizade, H. , & Ebadzade, H. R. (2019). Iran agriculture statistics, horticulture crops (Vol. 3). Ministry of Agriculture‐Jahad, Deputy of Planning and Economics, Information and Communication Technology Center.

[fsn32213-bib-0003] Amanatidou, A. , Slump, R. A. , Gorris, L. G. M. , & Smid, E. J. (2000). High oxygen and high carbon dioxide modified atmospheres for shelf‐life extension of minimally processed carrots. Journal of Food Science, 65(1), 61–66. 10.1111/j.1365-2621.2000.tb15956.x

[fsn32213-bib-0004] Arabpoor, B. , Yousefi, S. , Weisany, W. , & Ghasemlou, M. (2021). Multifunctional coating composed of *Eryngium campestre* L. essential oil encapsulated in nano‐chitosan to prolong the shelf‐life of fresh cherry fruits. Food Hydrocolloids, 111, 11. 10.1016/j.foodhyd.2020.106394

[fsn32213-bib-0005] Artés, F. , Villaescusa, R. , & Tudela, J. A. (2000). Modified atmosphere packaging of pomegranate. Journal of Food Science, 65(7), 1112–1116. 10.1111/j.1365-2621.2000.tb10248.x

[fsn32213-bib-0006] Ayhan, Z. , & Eştürk, O. (2009). Overall quality and shelf life of minimally processed and modified atmosphere packaged “ready‐to‐eat” pomegranate arils. Journal of Food Science, 74(5), C399–C405. 10.1111/j.1750-3841.2009.01184.x 19646034

[fsn32213-bib-0007] Ayub, H. , Ahmad, A. , Amir, R. M. , & Irshad, G. (2020). Multivariate analysis of peach quality treated with essential oil coatings. Journal of Food Processing and Preservation, 45, e15083. 10.1111/jfpp.15083

[fsn32213-bib-0008] Bagheri, R. , Izadi Amoli, R. , Tabari Shahndasht, N. , & Shahosseini, S. R. (2016). Comparing the effect of encapsulated and unencapsulated fennel extracts on the shelf life of minced common kilka (*Clupeonella cultriventris* caspia) and *Pseudomonas aeruginosa* inoculated in the mince. Food Sciences and Nutrition, 4(2), 216–222. 10.1002/fsn3.275 PMC4779485 27004111

[fsn32213-bib-0009] Banda, K. , Caleb, O. J. , Jacobs, K. , & Opara, U. L. (2015). Effect of active‐modified atmosphere packaging on the respiration rate and quality of pomegranate arils (cv. Wonderful). Postharvest Biology and Technology, 109, 97–105. 10.1016/j.postharvbio.2015.06.002

[fsn32213-bib-0010] Barros, L. , Ferreira, M.‐J. , Queirós, B. , Ferreira, I. C. F. R. , & Baptista, P. (2007). Total phenols, ascorbic acid, β‐carotene and lycopene in Portuguese wild edible mushrooms and their antioxidant activities. Food Chemistry, 103(2), 413–419. 10.1016/j.foodchem.2006.07.038

[fsn32213-bib-0011] Barzegar, H. , Jokar, A. , & Eslami, M. (2018). Effect of Persian gum coating containing cinnamon essential oil on the shelf life of pomegranate arils. Iranian Biosystem Engineering, 50(1), 67–76.(In Persian).

[fsn32213-bib-0012] Bhatia, K. , Asrey, R. , Jha, S. K. , Singh, S. , & Kannaujia, P. K. (2013). Influence of packaging material on quality characteristics of minimally processed Mridula pomegranate (*Punica granatum*) arils during cold storage. Indian Journal of Agricultural Sciences, 83(8), 872–876.

[fsn32213-bib-0013] Bhatia, K. , Asrey, R. , & Varghese, E. (2015). Correct packaging retained phytochemical, antioxidant properties and increases shelf life of minimally processed pomegranate (*Punica granatum* L.) arils Cv. Mridula. Journal of Scientific and Industrial Research, 74(3), 141–144.

[fsn32213-bib-0014] Burt, S. (2004). Essential oils: Their antibacterial properties and potential applications in foods—A review. International Journal of Food Microbiology, 94(3), 223–253. 10.1016/j.ijfoodmicro.2004.03.022 15246235

[fsn32213-bib-0015] Caleb, O. J. , Aindongo, W. V. , Opara, U. L. , & Mokwena, L. (2015). Effect of pre‐treatment and modified atmosphere packaging on quality attributes and volatile composition of pomegranate arils (‘Bhagwa’). Acta Horticulturae, 1079, 165–171. 10.17660/ActaHortic.2015.1079.17

[fsn32213-bib-0016] de Oliveira, L. I. G. , de Oliveira, K. Á. R. , de Medeiros, E. S. , Batista, A. U. D. , Madruga, M. S. , dos Santos Lima, M. , de Souza, E. L. , & Magnani, M. (2020). Characterization and efficacy of a composite coating containing chitosan and lemongrass essential oil on postharvest quality of guava. Innovative Food Science & Emerging Technologies, 66, 102506. 10.1016/j.ifset.2020.102506

[fsn32213-bib-0017] Dokhanieh, A. Y. , Aghdam, M. S. , & Sarcheshmeh, M. A. A. (2016). Impact of postharvest hot salicylic acid treatment on aril browning and nutritional quality in fresh‐cut pomegranate. Horticulture Environment and Biotechnology, 57(4), 378–384. 10.1007/s13580-016-0087-8

[fsn32213-bib-0018] Frazao, G. G. S. , Blank, A. F. , & de Aquino Santana, L. C. L. (2017). Optimisation of edible chitosan coatings formulations incorporating Myrcia ovata Cambessedes essential oil with antimicrobial potential against foodborne bacteria and natural microflora of mangaba fruits. LWT‐Food Science and Technology, 79, 1–10. 10.1016/j.lwt.2017.01.011

[fsn32213-bib-0019] Gardesh, A. S. K. , Badii, F. , Hashemi, M. , Ardakani, A. Y. , Maftoonazad, N. , & Gorji, A. M. (2016). Effect of nanochitosan based coating on climacteric behavior and postharvest shelf‐life extension of apple cv. Golab Kohanz. LWT‐Food Science and Technology, 70, 33–40. 10.1016/j.lwt.2016.02.002

[fsn32213-bib-0020] Ghasemnezhad, M. , Zareh, S. , Rassa, M. , & Sajedi, R. H. (2013). Effect of chitosan coating on maintenance of aril quality, microbial population and PPO activity of pomegranate (*Punica granatum* L. cv. Tarom) at cold storage temperature. Journal of the Science of Food and Agriculture, 93(2), 368–374. 10.1002/jsfa.5770 22821221

[fsn32213-bib-0021] Gil, M. , Martinez, J. , & Artes, F. (1996). Minimally processed pomegranate seeds. LWT ‐ Food Science and Technology, 29(8), 708–713. 10.1006/fstl.1996.0110

[fsn32213-bib-0022] Gniewosz, M. , Krasniewska, K. , Woreta, M. , & Kosakowska, O. (2013). Antimicrobial activity of a pullulan‐caraway essential oil coating on reduction of food microorganisms and quality in fresh baby carrot. Journal of Food Science, 78(8), M1242–M1248. 10.1111/1750-3841.12217 23957414

[fsn32213-bib-0023] Hashemi, S. M. B. , Mousavi Khaneghah, A. , Ghaderi Ghahfarrokhi, M. , & Eş, I. (2017). Basil‐seed gum containing *Origanum vulgare* subsp. viride essential oil as edible coating for fresh cut apricots. Postharvest Biology and Technology, 125, 26–34. 10.1016/j.postharvbio.2016.11.003

[fsn32213-bib-0024] Hussein, Z. , Caleb, O. J. , Jacobs, K. , Manley, M. , & Opara, U. L. (2015). Effect of perforation‐mediated modified atmosphere packaging and storage duration on physicochemical properties and microbial quality of fresh minimally processed “Acco” pomegranate arils. LWT ‐ Food Science and Technology, 64(2), 911–918. 10.1016/j.lwt.2015.06.040

[fsn32213-bib-0025] Javadian, S. R. , Shahosseini, S. R. , & Ariaii, P. (2017). The effects of liposomal encapsulated thyme extract on the quality of fish mince and *Escherichia coli* O157:H7 inhibition during refrigerated storage. Journal of Aquatic Food Product Technology, 26(1), 115–123. 10.1080/10498850.2015.1101629

[fsn32213-bib-0026] Jiang, Y. , & Li, Y. (2001). Effects of chitosan coating on postharvest life and quality of longan fruit. Food Chemistry, 73(2), 139–143. 10.1016/S0308-8146(00)00246-6

[fsn32213-bib-0027] Kapetanakou, A. E. , Stragkas, I. G. , & Skandamis, P. N. (2015). Developing an antimicrobial packaging of ready‐to‐eat pomegranate arils based on vapors of brandy or distillery ethanol. Food Research International, 69(1), 141–150. 10.1016/j.foodres.2014.12.006

[fsn32213-bib-0028] Khaliq, G. , Muda Mohamed, M. T. , Ali, A. , Ding, P. , & Ghazali, H. M. (2015). Effect of gum arabic coating combined with calcium chloride on physico‐chemical and qualitative properties of mango (*Mangifera indica* L.) fruit during low temperature storage. Scientia Horticulturae, 190, 187–194. 10.1016/j.scienta.2015.04.020

[fsn32213-bib-0029] Khorram, F. , Ramezanian, A. , & Hosseini, S. M. H. (2017). Shellac, gelatin and Persian gum as alternative coating for orange fruit. Scientia Horticulturae, 225, 22–28. 10.1016/j.scienta.2017.06.045

[fsn32213-bib-0030] Kumar, S. , Kumar, R. , & Nambi, V. E. (2016). Effect of pectin methyl esterase and Ca2+ ions treatment on antioxidant capacity, shelf‐life and quality of minimally processed Pomegranate (*Punica granatum* L.) arils. Journal of Environmental Biology, 37(2), 193–199. 27097437

[fsn32213-bib-0031] Lawless, H. T. , & Heymann, H. (2010). Sensory evaluation of food: Principles and practices. Springer.

[fsn32213-bib-0032] López‐Rubira, V. , Conesa, A. , Allende, A. , & Artés, F. (2005). Shelf life and overall quality of minimally processed pomegranate arils modified atmosphere packaged and treated with UV‐C. Postharvest Biology and Technology, 37(2), 174–185. 10.1016/j.postharvbio.2005.04.003

[fsn32213-bib-0033] Maftoonazad, N. , & Ramaswamy, H. S. (2019). Application and evaluation of a pectin‐based edible coating process for quality change kinetics and shelf‐life extension of lime fruit (Citrus aurantifolium). Coatings, 9(5), 285. 10.3390/coatings9050285

[fsn32213-bib-0034] Martínez‐Abad, A. , Sánchez, G. , Fuster, V. , Lagaron, J. M. , & Ocio, M. J. (2013). Antibacterial performance of solvent cast polycaprolactone (PCL) films containing essential oils. Food Control, 34(1), 214–220. 10.1016/j.foodcont.2013.04.025

[fsn32213-bib-0035] Martínez‐Romero, D. , Castillo, S. , Guillén, F. , Díaz‐Mula, H. M. , Zapata, P. J. , Valero, D. , & Serrano, M. (2013). Aloe vera gel coating maintains quality and safety of ready‐to‐eat pomegranate arils. Postharvest Biology and Technology, 86, 107–112. 10.1016/j.postharvbio.2013.06.022

[fsn32213-bib-0036] Min, T. , Sun, X. , Yuan, Z. , Zhou, L. , Jiao, X. , Zha, J. , Zhu, Z. , & Wen, Y. (2021). Novel antimicrobial packaging film based on porous poly(lactic acid) nanofiber and polymeric coating for humidity‐controlled release of thyme essential oil. LWT‐Food Science and Technology, 135, 8. 10.1016/j.lwt.2020.110034

[fsn32213-bib-0037] Moalemiyan, M. , Ramaswamy, H. S. , & Maftoonazad, N. (2012). Pectin‐based edible coating for shelf life extension of ataulfo mango. Journal of Food Process Engineering, 35(4), 572–600. 10.1111/j.1745-4530.2010.00609.x

[fsn32213-bib-0038] Moreira, M. R. , Tomadoni, B. , Martín‐Belloso, O. , & Soliva‐Fortuny, R. (2015). Preservation of fresh‐cut apple quality attributes by pulsed light in combination with gellan gum‐based prebiotic edible coatings. LWT ‐ Food Science and Technology, 64(2), 1130–1137. 10.1016/j.lwt.2015.07.002

[fsn32213-bib-0039] Mostafavi, F. S. (2019). The surface characteristics of biopolymer‐coated tomato and cucumber epicarps: Effect of guar, Persian and tragacanth gums. Journal of Food Measurement and Characterization, 13(1), 840–847. 10.1007/s11694-018-9996-9

[fsn32213-bib-0040] Motelica, L. , Ficai, D. , Ficai, A. , Trusca, R. D. , Ilie, C. I. , Oprea, O. C. , & Andronescu, E. (2020). Innovative antimicrobial chitosan/zno/ag nps/citronella essential oil nanocomposite ‐ Potential coating for grapes. Foods, 9(12), 26. 10.3390/foods9121801 PMC7761909 33291604

[fsn32213-bib-0041] O', L. , Grady, N. A. , Sigge, G. O. , Caleb, O. J. , & Opara, U. L. (2015). Effects of water dipping of whole fruit on the microbial quality of minimally processed pomegranate (*Punica granatum* L.) arils during cold storage. International Journal of Postharvest Technology and Innovation, 5(1), 1–7. 10.1504/IJPTI.2015.072435

[fsn32213-bib-0042] Oz, A. T. , & Ulukanli, Z. (2012). Application of edible starch‐based coating including glycerol plus oleum Nigella on arils from long‐stored whole pomegranate fruits. Journal of Food Processing and Preservation, 36(1), 81–95. 10.1111/j.1745-4549.2011.00599.x

[fsn32213-bib-0043] Palma, A. , Continella, A. , Malfa, S. L. , Gentile, A. , & D'Aquino, S. (2015). Overall quality of ready‐to‐eat pomegranate arils processed from cold stored fruit. Postharvest Biology and Technology, 109, 1–9. 10.1016/j.postharvbio.2015.06.001

[fsn32213-bib-0044] Peña‐Estévez, M. E. , Artés‐Hernández, F. , Artés, F. , Aguayo, E. , Martínez‐Hernández, G. B. , Galindo, A. , & Gómez, P. A. (2016). Quality changes of pomegranate arils throughout shelf life affected by deficit irrigation and pre‐processing storage. Food Chemistry, 209, 302–311. 10.1016/j.foodchem.2016.04.054 27173567

[fsn32213-bib-0045] Rahimi, S. , Abbasi, S. , Sahari, M. A. , & Azizi, M. H. (2013). Separation and determination of some chemical and functional properties of soluble and insoluble fractions of mountain almond tree gum (Persian gum). Iranian Food Science and Technology, 10(40), 1–10.(In Persian).

[fsn32213-bib-0046] Roller, S. , & Seedhar, P. (2002). Carvacrol and cinnamic acid inhibit microbial growth in fresh‐cut melon and kiwifruit at 4 ^o^C and 8 ^o^C. Letters in Applied Microbiology, 35, 5. 10.1046/j.1472-765x.2002.01209.x 12390487

[fsn32213-bib-0047] Salehi, F. (2020). Edible coating of fruits and vegetables using natural gums: A review. International Journal of Fruit Science, 20, S570–S589. 10.1080/15538362.2020.1746730

[fsn32213-bib-0048] Samari‐Khalaj, M. , & Abbasi, S. (2017). Solubilisation of Persian gum: Chemical modification using acrylamide. International Journal of Biological Macromolecules, 101, 187–195. 10.1016/j.ijbiomac.2017.03.046 28286077

[fsn32213-bib-0049] Shaygannia, S. , Eshaghi, M. R. , Fazel, M. , & Hashemiravan, M. (2020). Phenolic compounds and antioxidant activities of lemon wastes affected by microencapsulation using coatings of Arabic, Persian, and basil seed gums. Journal of Food Measurement and Characterization, 11, 1–11. 10.1007/s11694-020-00732-6

[fsn32213-bib-0050] Slinkard, K. , & Singleton, V. L. (1977). Total phenol analysis: Automation and comparison with manual methods. American Journal of Enology and Viticulture, 28, 49–55.

[fsn32213-bib-0051] Soliva‐Fortuny, R. C. , Grigelmo‐Miguel, N. , Hernando, I. , Lluch, M. Á. , & Martín‐Belloso, O. (2002). Effect of minimal processing on the textural and structural properties of fresh‐cut pears. Journal of the Science of Food and Agriculture, 82(14), 1682–1688. 10.1002/jsfa.1248

[fsn32213-bib-0052] Tahir, H. E. , Zou, X. B. , Mahunu, G. K. , Arslan, M. , Abdalhai, M. , & Li, Z. H. (2019). Recent developments in gum edible coating applications for fruits and vegetables preservation: A review. Carbohydrate Polymers, 224, 115141. 10.1016/j.carbpol.2019.115141 31472839

[fsn32213-bib-0053] Varasteh, F. , Arzani, K. , Barzegar, M. , & Zamani, Z. (2012). Changes in anthocyanins in arils of chitosan‐coated pomegranate (*Punica granatum* L. cv. Rabbab‐e‐Neyriz) fruit during cold storage. Food Chemistry, 130(2), 267–272. 10.1016/j.foodchem.2011.07.031

[fsn32213-bib-0054] Villafañe, F. (2017). Edible coatings for carrots. Food Reviews International, 33(1), 84–103. 10.1080/87559129.2016.1150291

[fsn32213-bib-0055] Wang, W. X. , Zhang, Y. L. , Yang, Z. , & He, Q. (2021). Effects of incorporation with clove (Eugenia caryophyllata) essential oil (CEO) on overall performance of chitosan as active coating. International Journal of Biological Macromolecules, 166, 578–586. 10.1016/j.ijbiomac.2020.10.215 33137383

[fsn32213-bib-0056] Yousuf, B. , & Srivastava, A. K. (2015). Psyllium (Plantago) gum as an effective edible coating to improve quality and shelf life of fresh‐cut papaya (*Carica papaya*). World Academy of Science, Engineering and Technology, International Journal of Nutrition and Food Engineering, 2, 765–770.

[fsn32213-bib-0057] Zahran, A. A. , Hassanein, R. A. , & AbdelWahab, A. T. (2015). Effect of chitosan on biochemical composition and antioxidant activity of minimally processed ‘Wonderful’ pomegranate arils during cold storage. Journal of Applied Botany and Food Quality, 88, 241–248. 10.5073/JABFQ.2015.088.035

